# Nonlinear parameters of surface EMG in schizophrenia patients depend on kind of antipsychotic therapy

**DOI:** 10.3389/fphys.2015.00197

**Published:** 2015-07-10

**Authors:** Alexander Yu. Meigal, German G. Miroshnichenko, Anna P. Kuzmina, Saara M. Rissanen, Stefanos D. Georgiadis, Pasi A. Karjalainen

**Affiliations:** ^1^Laboratory for Novel Methods in Physiology, Institute of High-Tech Biomedical Solutions, Petrozavodsk State UniversityPetrozavodsk, Russia; ^2^Biosignal Analysis and Medical Imaging Group, Department of Applied Physics, Faculty of Science and Forestry, University of Eastern FinlandKuopio, Finland; ^3^Republican Psychiatric HospitalMatrosy, Russia

**Keywords:** surface EMG, spectrum, recurrence quantification, mutual information, schizophrenia, antipsychotics, parkinsonism, motor symptoms

## Abstract

We compared a set of surface EMG (sEMG) parameters in several groups of schizophrenia (SZ, *n* = 74) patients and healthy controls (*n* = 11) and coupled them with the clinical data. sEMG records were quantified with spectral, mutual information (MI) based and recurrence quantification analysis (RQA) parameters, and with approximate and sample entropies (ApEn and SampEn). Psychotic deterioration was estimated with Positive and Negative Syndrome Scale (PANSS) and with the positive subscale of PANSS. Neuroleptic-induced parkinsonism (NIP) motor symptoms were estimated with Simpson-Angus Scale (SAS). Dyskinesia was measured with Abnormal Involuntary Movement Scale (AIMS). We found that there was no difference in values of sEMG parameters between healthy controls and drug-naïve SZ patients. The most specific group was formed of SZ patients who were administered both typical and atypical antipsychotics (AP). Their sEMG parameters were significantly different from those of SZ patients taking either typical or atypical AP or taking no AP. This may represent a kind of synergistic effect of these two classes of AP. For the clinical data we found that PANSS, SAS, and AIMS were not correlated to any of the sEMG parameters. Conclusion: with nonlinear parameters of sEMG it is possible to reveal NIP in SZ patients, and it may help to discriminate between different clinical groups of SZ patients. Combined typical and atypical AP therapy has stronger effect on sEMG than a therapy with AP of only one class.

## 1. Introduction

Schizophrenia (SZ) is one of the most serious and intensively studied neuropsychiatric disorders. About 1% of population suffers from schizophrenia, regardless of the development level of a country (Tandon et al., 2008). SZ is characterized by distinct phasic course, with a premorbid, a prodromal, a psychotic phase, and a phase of remission (Tandon et al., 2009). The overall economic burden of SZ, for example in the USA, was estimated to be $62.7 billion in 2002 (Wu et al., 2005).

According to the plausible current hypothesis, SZ symptoms are associated with increased dopamine production in mesocortical and mesolimbic pathways and increased density of D2-receptors (van Os and Kapur, 2009). Dopamine receptors play an important part in muscle tone regulation (Double and Crocker, 1995), so it is likely that hyperproduction of dopamine and increased sensitivity of dopamine receptors in SZ can affect not only mental, but also the motor processes. Indeed, patients with SZ exhibit a variety of motor deficits. Among them are ataxia, extrapyramidal disturbances, pathological locomotor patterns (Goryunova, 1994), dyskinesia (Puri et al., 1999), asymmetry of motoneurons excitability (Goode and Manning, 1988), reduced ability to motor reorganization, and disrupted neural plasticity (Daskalakis et al., 2008). A recent study found that such hyperkinetic movements as writhing or flinging movements of the limbs, fingers or face indicated those high-risk individuals who would later convert to psychosis (Mittal and Walker, 2007). Electromyography (EMG) is believed to be a good instrumental method to ascertain whether specific dyskinesias (e.g., dystonia) or dyskinesias of specific body regions are associated with transitioning to psychosis (Callaway et al., 2014). Earlier, Crayton et al. (1977) have revealed increased muscle fiber density and number of single muscle fiber action potentials belonging to the same motor unit (MU), thus evidencing denervation/reinnervation process in psychotic patients. (Flyckt et al., 2000) have revealed in SZ patients pathologically increased amplitude of MU action potentials, which nonetheless did not correlate with muscle biopsy findings.

Interestingly, in Parkinson's disease (PD), which is characterized by the dopamine deficiency in the basal ganglia, surface EMG (sEMG) contains large portions of clustered potentials. This causes specific changes in many of sEMG nonlinear parameters, e.g., decreased entropy and correlation dimension, and increased percent of determinism. sEMG in PD in a way is more predictable, less complex, and more regular (Meigal et al., 2009, 2012). Such pattern of sEMG may be caused by increased synchronization of motor units (Fattorini et al., 2005). Also, the nonlinear parameters of sEMG were shown to be sensitive to the state of muscle pain (Sung et al., 2007). Psychosis in PD is a frequent condition affecting as much as 20% of all PD patients (Levin et al., 2015), while drug-induced parkinsonism (DIP) is seen in one third of patients exposed to antipsychotic drugs (Blanchet et al., 2012). Recently, we have demonstrated that most of sEMG parameters in a group of SZ patients were clearly different from the control group probably due to DIP (Miroshnichenko et al., 2014). We believe that coupling of clinical data from SZ patients with sEMG parameters would be helpful in further understanding of motor symptoms and sEMG changes in SZ patients.

Therefore, the aim of the present study was to couple a variety of sEMG parameters in several clinical groups of SZ patients with the positive and negative symptoms of SZ with special regard to the antipsychotic therapy.

## 2. Materials and methods

### 2.1. Patients

**Figure 1:**
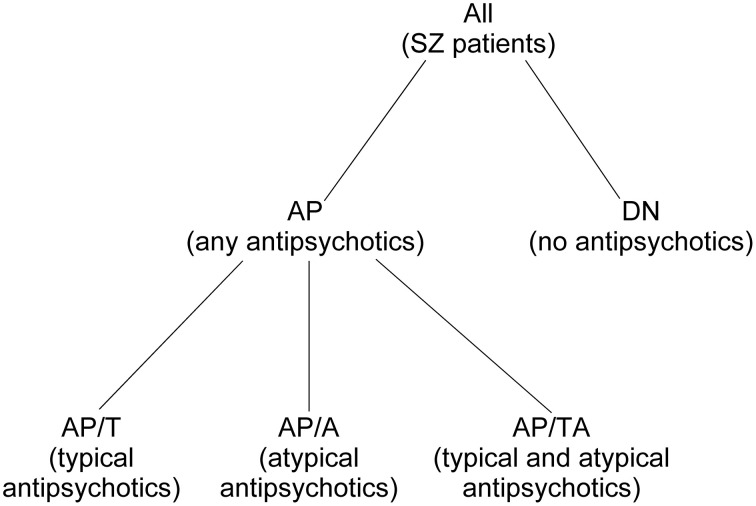
**Patients grouping based on their medication**.

We divided all SZ patients (*n* = 74, 18–58 years) in two groups: the SZ patients in remission phase (R group) and the patients in acute psychosis (P group). Also, we divided all SZ patients into the group of patients who were taking any antipsychotics at the moment of the study (AP group) and the group of drug-naïve patients who had never taken antipsychotics (DN). The AP group was split into the subgroup of patients who were taking typical antipsychotics (AP/T), the subgroup of patients who were taking atypical antipsychotics (AP/A), and the subgroup of patients who were taking both typical and atypical antipsychotics (AP/TA). See Figure 1 for the diagram of medication-based patients grouping. SZ phase and medication allowed to distinguish 17 non-empty groups of patients. See the groups sizes in Table 1. We also used Table 1 for notation of subgroups of the patients. Each subgroup was denoted with the names of its row and column in Table 1, e.g., R-AP/T is the subgroup of patients who were in remission and taking typical antipsychotics at the moment of study.

**Table 1:** **SZ patients groups sizes (total group size\PANSS is estimated\SAS and AIMS are estimated)**.

**Group**	**All**	**AP**	**AP/T**	**AP/A**	**AP/TA**	**DN**
All	74\58\62	63\49\54	25\20\21	24\19\22	14\10\11	11\9\8
R	13\10\11	13\10\11	7\6\7	3\2\2	3\2\2	0
P	61\48\51	50\39\43	18\14\14	21\17\20	11\8\9	11\9\8

The SZ patients in acute psychosis were examined in the Republican Psychiatric Hospital (settlement Matrosy, Republic of Karelia, Russian Federation). The SZ patients in remission phase were examined in the Republican Psychoneurologic Dispensary (Petrozavodsk, Russian Federation). A variety of symptoms, such as delusions, hallucinations, psychomotor stupor or agitation, disorganized behavior, and absence of criticism of pathological experience were characteristic for all patients under psychotic condition. Duration of psychosis by examination ranged from several days to several weeks. Some patients had catatonia, which mainly appeared in the form of substupor with lethargy, catalepsy, mutism, and usually it was combined with delusions or hallucinations. See the medication of the patients in Table 2. Examination of the antipsychotic-naïve patients as a separate group (DN) seems to be a relevant model to investigate the disease *per se* and to evaluate the action of antipsychotic medication. The R group was formed of patients without psychotic deterioration for at least 6 months. The patients who had organic brain lesions beside schizophrenia (traumatic brain injury, neuroinfections, alcoholism, substance dependencies, and vascular brain diseases) were excluded from the study, as well as the patients who were taking psychotropic drugs beside antipsychotics (antidepressants, mood-stabilizing drugs) or drug-induced parkinsonism correctors such as trihexyphenidyl. Taking of benzodiazepine tranquilizers was accepted not less than 15 hours before examination. A control group (Con, *n* = 11) was formed of mentally and physically healthy subjects (20–40 years) who were age- and sex-matched to the DN group.

**Table 2:** **Medication of the patients**.

**No**.	**Group**	**Medication**
1	P-AP/T	zuclopenthixol (tf) 30 mg DD
2	P-AP/A	clozapine (to) 300 mg DD
3	P-AP/TA	clozapine (to) 300 mg DD,
		chlorpromazine HCl (to) 300 mg DD
4	P-AP/A	clozapine (to) 50 mg DD
5	P-AP/T	zuclopenthixol acetate (im) 100 mg AD,
		chlorpromazine HCl (pl) 300 mg DD
6	P-AP/A	quetiapine fumarate (tf) 800 mg DD
7	P-AP/A	risperidone (tf) 6 mg DD
8	P-AP/A	quetiapine fumarate (te) 600 mg DD,
		clozapine (to) 25 mg DD
9	P-AP/A	risperidone (so) 8 mg DD
10	P-AP/A	clozapine (to) 500 mg DD
11	P-AP/T	zuclopenthixol decanoate (im) 200 mg 3W,
		periciazine (cp) 30 mg DD
12	P-AP/T	haloperidol (im) 35 mg DD,
		chlorpromazine HCl (im) 100 mg DD
13	P-AP/A	risperidone (so) 4 mg DD
14	P-AP/A	risperidone (td) 8 mg DD
15	P-AP/A	risperidone (td) 6 mg DD
16	P-AP/A	risperidone (tf) 3 mg DD
17	P-AP/TA	risperidone (tf) 10 mg DD, clozapine (to)
		100 mg DD, haloperidol (to) 20 mg DD
18	P-AP/A	risperidone (tc) 2 mg DD,
		clozapine (to) 150 mg DD
19	P-AP/TA	olanzapine (tc) 15 mg DD,
		chlorpromazine HCl (im) 50 mg DD
20	P-AP/A	risperidone (tf) 6 mg DD
21	P-AP/T	haloperidol (im) 20 mg DD,
		chlorpromazine HCl (im) 50 mg DD
22	P-AP/T	haloperidol (im) 15 mg DD
23	P-AP/T	haloperidol (im) 10 mg DD
24	P-AP/A	quetiapine fumarate (te) 400 mg DD
25	P-AP/T	haloperidol (im) 10 mg DD
26	P-AP/A	risperidone (so) 6 mg DD
27	P-AP/T	haloperidol (im) 30 mg DD,
		chlorpromazine HCl (im) 50 mg DD
28	P-AP/T	haloperidol (im) 20 mg DD
29	P-AP/T	zuclopenthixol (tf) 40 mg DD
30	P-AP/TA	haloperidol (to) 45 mg DD,
		olanzapine (tc) 15 mg DD
31	P-AP/A	risperidone (ie) 37.5 mg 2W
32	P-AP/TA	clozapine (to) 175 mg DD,
		haloperidol (to) 30 mg DD
33	P-AP/TA	haloperidol (to) 5 mg DD,
		clozapine (to) 300 mg DD
34	R-AP/A	sertindole (tc) 4 mg DD
35	R-AP/T	chlorpromazine HCl (pl) 100 mg DD,
		haloperidol decanoate (im) 50 mg 4W
36	R-AP/TA	trifluoperazine (tc) 30 mg DD,
		clozapine (to) 250 mg DD
37	P-AP/T	haloperidol (im) 10 mg DD
38	P-AP/A	risperidone (tf) 8 mg DD
39	P-AP/TA	haloperidol (im) 30 mg DD,
		clozapine (to) 300 mg DD
40	P-AP/T	haloperidol (im) 10 mg DD
41	P-AP/TA	haloperidol (to) 30 mg DD,
		clozapine (to) 100 mg DD
42	P-AP/TA	haloperidol (im) 20 mg DD,
		clozapine (to) 100 mg DD
43	P-AP/T	zuclopenthixol acetate (im) 100 mg AD
44	P-AP/T	haloperidol (im) 5 mg DD
45	P-AP/A	quetiapine fumarate (te) 600 mg DD
46	R-AP/T	haloperidol decanoate (im) 50 mg 3W
47	P-AP/T	haloperidol (im) 10 mg DD,
		chlorpromazine HCl (im) 50 mg DD
48	R-AP/T	haloperidol decanoate (im) 50 mg 4W
49	R-AP/T	trifluoperazine (tc) 5 mg DD,
		chlorprothixene (tf) 50 mg DD
50	R-AP/TA	haloperidol decanoate (im) 100 mg 2W,
		clozapine (to) 100 mg DD
51	R-AP/TA	haloperidol decanoate (im) 50 mg 4W,
		clozapine (to) 100 mg DD
52	R-AP/T	zuclopenthixol decanoate (im) 200 mg 4W
53	R-AP/T	fluphenazine decanoate (im) 25 mg 3W,
		levomepromazine (tc) 25 mg DD
54	P-AP/A	risperidone (so) 3 mg DD
55	P-AP/T	haloperidol (im) 5 mg DD
56	P-AP/TA	haloperidol (to) 45 mg DD,
		clozapine (to) 550 mg DD
57	P-AP/T	zuclopenthixol acetate (im) 50 mg AD
58	P-AP/A	risperidone (so) 3 mg DD
59	P-AP/TA	clozapine (to) 100 mg DD,
		haloperidol (to) 15 mg DD
60	P-AP/A	risperidone (tf) 4 mg DD,
		clozapine (to) 6.25 mg DD
61	R-AP/A	clozapine (to) 200 mg DD
62	R-AP/A	olanzapine (tf) 10 mg DD
63	R-AP/T	fluphenazine decanoate (im) 25 mg 4W

*DD, daily; AD, on alternate days; 2W/3W/4W, every 2/3/4 weeks; to, tablet; tc, tablet, coated; tf, tablet, film coated; te, tablet, film coated, extended release; td, tablet, orally disintegrating; pl, pill; cp, capsule; im, injection, solution, intramuscular; ie, injection, suspension, extended release, intramuscular; so, solution, oral*.

The study was approved by the ethical committee of the Republican Psychiatric Hospital (decision #8, 4/11/2012). Written informed consents were obtained from all patients and healthy control subjects.

### 2.2. Methods

Psychotic deterioration of 58 patients (see their groups in Table 1) was estimated with Positive and Negative Syndrome Scale (PANSS, Kay et al., 1987), with a separate consideration of the positive subscale of PANSS (PSS). Drug-induced parkinsonism (DIP) motor symptoms of 62 patients (see their groups in Table 1) were estimated with Simpson-Angus Scale (SAS, Simpson and Angus, 1970). Dyskinesia of 62 patients (the same as for SAS) was measured with Abnormal Involuntary Movement Scale (AIMS, Guy, 1976).

sEMG were measured in standing position from *m. biceps brachii* with the forearm flexed at 90° (parallel to floor) and the palm opened and directed upwards. Prior to electrode placement, the skin was carefully cleaned with cotton alcohol swab. A bipolar plate-mounted electrode with inter-electrode distance of 15 mm was used. The reference electrode was attached to ipsilateral wrist. The records were performed with Neuro-MEP-4 (NeuroSoft Inc., Ivanovo, Russian Federation) at sampling frequency 20 kHz with 50 Hz notch filter enabled. The lower and upper cutoff frequencies of the filter of the amplifier of the measuring device were set to 50 and 1000 Hz respectively (−3 dB attenuation at cutoff frequencies, frequency responses are the same as for 1st order high-pass and 2nd order low-pass Butterworth filters). For each subject, first record was made without any load, then three more records were made with 1, 2, and 3 kg loads on the palm. Each record was 1 s (20001 samples) long. Data processing and analysis, with the exception of mutual information calculations, were performed with Matlab software (MathWorks Inc., Natick, USA). Mutual information was computed using program by (Weeks, 1997).

Five measurements from the SZ patients and one measurement from the Con group appeared to have artifacts in the sEMG measured with some of the loads, so those records were excluded from the study (the number of patients, group, loads: 1 P-AP/T 3 kg, 1 P-AP/A 0 kg, 1 P-DN 2,3 kg, 2 R-AP/TA 3 kg). The other 80 measurements were used completely.

A visual check of the sEMG spectra revealed that the spectra of some records had sharp peaks at frequencies multiple of 50 Hz, so we applied Fourier interpolation suggested by (Mewett et al., 2004) to all the records. The amplitudes at frequencies multiple of 50 Hz in the range 50–400 Hz were replaced by linearly interpolated values calculated from two adjacent points (±1 Hz), and the phases were kept the same. Then the records were low-pass filtered with the 14th order elliptic filter designed with Matlab design function (passband frequency 420 Hz, stopband frequency 500 Hz, passband ripple 2· 10^−6^ dB, stopband attenuation 80 dB, passband exact match). Then the signals were detrended with smoothness priors method introduced by (Tarvainen et al., 2002) to remove the movement artifacts those might remain after high-pass filtering by the device. Smoothness priors detrending method can be viewed as a time-varying FIR high-pass filter, which causes less attenuation in the beginning and the end of a signal. This method can be used for trend estimation or removal of the trend by subtracting it from the signal. The only detrending parameter λ affects the trend smoothness. Larger λ values correspond to more smooth trends and lower cutoff frequencies. We used λ_*detr*_ = 10^5^, which corresponds to attenuation of –40 dB at 10 Hz in the middle of a signal.

We calculated mean and median frequencies of amplitude and power FFT spectra (MNF_A_, MDF_A_, MNF_P_, MDF_P_). The signals were windowed with Hanning window before the calculations to eliminate the spectrum distortion caused by discontinuities at the beginning and the end of a signal.

A phase space embedding procedure was performed for the further parameters calculations. Signal values {*x*_1_, *x*_2_, …, *x_n_*} were replaced by vectors *X_i_* in *m*-dimensional space (Equation 1), with *L* being the time lag.

(1) $$X_{i}=(x_{i},\;x_{i+L},\;x_{i+2L},\;\ldots ,\;x_{i+(m-1)L})$$

If *m* and *L* values are correct, then *X_i_* values make up a smooth trajectory in ℝ^*m*^ space, which corresponds to the dynamics of the examined system. Mathematical justification of this procedure is the embedding theorem by Takens (1981) and Mañé (1981). *L* was chosen with the help of mutual information as recommended by (Fraser and Swinney, 1986), and (Celucci et al., 2003). For each signal we calculated mutual information *I* of the signal and the same signal shifted backwards in time on τ sampling periods with τ varying from 1 to 100. The dependence *I*(τ) was calculated using the program of (Weeks, 1997). Since *I*(τ) dependencies were noisy, they were replaced by their trends obtained using smoothness priors method with λ_*tr*_ = 9.1. The first minimum *T* of an *I*(τ) trend was used as a separate sEMG parameter, and the optimal time lag *L* was chosen as mean *T* (*L* = 45). The smoothness priors parameter λ_*tr*_ = 9.1 was chosen from the most wide range where λ did not affect *T* of any record. Namely, we determined the dependence *T*(λ) for each record and obtained that λ > 15.531 gave strictly decreasing trends for some records, and 8.985 ≤ λ ≤ 9.144 was the most wide range that gave stable *T* for all the records. The embedding dimension *m* was chosen with the help of false nearest neighbors (FNN) method as advised by (Celucci et al., 2003) and (Kennel et al., 1992). We calculated the number of FNNs for each record with *m* varying from 1 to 15 and normalized the numbers by the number of FNNs for *m* = 1 multiplied by 100%. Minimum *m* that gave not more that 1% of FNNs for any record was chosen as the embedding dimension (*m* = 5).

Then approximate entropy (ApEn, Pincus, 1991) and sample entropy (SampEn, Richman and Moorman, 2000) were calculated using Euclidian interpoint distances and the tolerance distances *r_ApEn_* = 0.65 and *r_SampEn_* = 0.8756. The records were normalized to unit standard deviation. *r_ApEn_* and *r_SampEn_* were fitted to maximize Shannon entropies of the ApEn and SampEn distributions correspondingly. Each of the two distributions consisted of 334 bins ranging from ApEn_min_ to ApEn_max_ and from SampEn_min_ to SampEn_max_ correspondingly.

Further analysis was performed by means of recurrence plots described by (Marwan et al., 2007). Recurrence plot Rec is a matrix of size *N* × *N*, where *N* is the number of trajectory points. Matrix element at the crossing of a column and a row is equal to one (black point), if the points corresponding to the column and the row are not further apart from each other than ε, and is equal to zero (white point) otherwise as in Equation (2), where *i, j* = 1 … *N*, Θ(•) is the Heaviside function, ||*X*|| is Euclidean norm.

(2) $$Rec_{i,j}=\Theta (\varepsilon \;-\;\|X_{i}-X_{j}\|)$$

Recurrence plot makes it possible to analyze how often and for how long time a system returns to its previous states. As consecutive trajectory points are almost always close to each other, their proximity is not informative, so pairs of points with index difference less than *W* should not be considered. If *W* > 0, recurrence plot includes white strip along main diagonal, with horizontal width 1 + 2(*W* − 1) points. Then recurrence quantification analysis (RQA, Marwan et al., 2007) of the recurrence plots was performed. We calculated RR (share of black points of Rec), determinism DET (share of black points that form diagonal lines), laminarity LAM (share of black points that form vertical lines), RATIO = DET/RR, average diagonal line length L, average vertical line length (trapping time) TT, maximum diagonal line length L_max_, maximum vertical line length V_max_, divergence DIV = 1/L_max_, Shannon entropy of diagonal lines lengths probability distribution ENTR, and TREND. Key parameters *l_min_* and *v_min_* are required to calculate DET, LAM, L, TT, and ENTR. We fitted the key parameters ε, *W*, and *l_min_* to maximize sensitivity of DET to the load, namely we maximized sum of absolute values of Kendall's correlation coefficients of dependencies of DET on load among all subjects but the one with 2 and 3 kg records excluded (*W* = 6, *l_min_* = 3, ε = 0.09 · E||X_i_ − X_j_||, *i* ≠ *j*, where E means mean, and the mean is calculated using all the points pairs, including those with index difference less than *W*). *v_min_* was fitted to maximize similar sum of correlation coefficients for LAM given ε and *W* chosen with the help of DET (*v_min_* = 3). Parameter Ñ required for measure TREND was chosen as *N* − 10 (Ñ = 19811).

The order of statistical analysis is stated in Table 3. Kolmogorov-Smirnov test showed that the distribution of the sEMG parameters was not normal in almost all of the subjects groups, so we chose Kruskal–Wallis and Mann–Whitney tests for comparisons of the sEMG parameters between the groups. First we compared sEMG parameters between the eight indivisible subgroups P-AP/T, P-AP/A, P-AP/TA, R-AP/T, R-AP/A, R-AP/TA, P-NAP/DN, and Con using Kruskal–Wallis test (H0 in Table 3). The comparison was made for each sEMG parameter and each load separately (18 · 4 tests). Then hypotheses H1–H10 from Table 3 were tested using Mann-Whithey test only for the parameters and loads those were significantly different in the eight indivisible subgroups (*p* < 0.05 in H0 test). Bonferroni correction was applied to the significance level in the H1–H10 tests, namely only hypotheses with *p* < 0.05/10 were rejected. Clinical scales SAS and AIMS were compared between the All-AP and All-DN groups (H11–H12 in Table 3). We calculated Kendall's τ and Spearman's ρ correlation coefficients between the clinical scales and the sEMG parameters for each load separately. Correlation between the sEMG parameters and PANSS and PSS was calculated for the All-All group (H13–H14 in Table 3). Correlation between the sEMG parameters and SAS and AIMS was calculated for the All-AP group (H15–H16 in Table 3).

**Table 3:** **Null hypotheses and the groups of subjects used in corresponding statistical tests**.

**No**.	**Hypothesis**	**Subjects groups**
**H0**	sEMG parameters have the same distributions in the groups P-AP/T, P-AP/A, P-AP/TA, R-AP/T, R-AP/A, R-AP/TA, P-NAP/DN, Con.	–
H1	Psychosis state does not influence sEMG parameters.	Con and P-NAP/DN
H2	Provided psychosis state, taking of typical AP at the moment of study does not influence sEMG parameters.	P-AP/T and P-NAP/DN
H3	Provided psychosis state, taking of atypical AP at the moment of study does not influence sEMG parameters.	P-AP/A and P-NAP/DN
**H4**	Provided psychosis state, taking of both typical and atypical AP at the moment of study does not influence sEMG parameters.	P-AP/TA and P-NAP/DN
H5	Provided psychosis state, taking of typical AP at the moment of study influences sEMG parameters in the same way as taking of atypical AP at the moment of study.	P-AP/T and P-AP/A
**H6**	Provided psychosis state, taking of typical AP at the moment of study influences sEMG parameters in the same way as taking of both typical and atypical AP at the moment of study.	P-AP/T and P-AP/TA
**H7**	Provided psychosis state, taking of atypical AP at the moment of study influences sEMG parameters in the same way as taking of both typical and atypical AP at the moment of study.	P-AP/A and P-AP/TA
H8	Provided remission state, taking of typical AP at the moment of study influences sEMG parameters in the same way as taking of atypical AP at the moment of study.	R-AP/T and R-AP/A
H9	Provided remission state, taking of typical AP at the moment of study influences sEMG parameters in the same way as taking of both typical and atypical AP at the moment of study.	R-AP/T and R-AP/TA
H10	Provided remission state, taking of atypical AP at the moment of study influences sEMG parameters in the same way as taking of both typical and atypical AP at the moment of study.	R-AP/A and R-AP/TA
**H11**	Taking of antipsychotics at the moment of study does not influence SAS	All-AP and All-DN
**H12**	Taking of antipsychotics at the moment of study does not influence AIMS	All-AP and All-DN
H13	sEMG parameters do not correlate to PANSS	All-All
H14	sEMG parameters do not correlate to PSS	All-All
H15	Provided taking of any antipsychotics at the moment of study, sEMG parameters do not correlate to SAS	All-AP
H16	Provided taking of any antipsychotics at the moment of study, sEMG parameters do not correlate to AIMS	All-AP

*Rejected null hypotheses are marked with boldface*.

Then the dimensionality of the data was reduced by means of principal component analysis (PCA, Jolliffe, 2002). The essence of PCA is to represent a set of feature vectors as a weighed sum of basis vectors, with weights being the principal components. We formed feature vectors *z^T^* (Equation 3) from sEMG parameters those were statistically significantly different in any of H1–H10 tests at 1 kg load. Each parameter was normalized to mean and standard deviation of its values.

(3) $$z^{T}=[\text{SampEn DET L TT ENTR}]$$

Then we obtained an orthonormal set of basis vectors, which represent directions of maximum variance in a space of the sEMG parameters included in *z^T^*. The basis vectors were solved using experimental correlation matrix obtained from the parameters for all subjects. We formed matrix *Z* from rows *z^T^_i_* of the sEMG parameters, each corresponding to the *i*-th subject, and calculated correlation matrix *R* as in Equation (4), where *N_s_* is the number of subjects.

(4) $$R=\frac{1}{N_{s}}Z^{T}Z$$

Then we obtained eigenvectors and eigenvalues of *R*. The eigenvectors were used as basis vectors. We sorted the basis vectors in decreasing order of their corresponding eigenvalues and formed matrix *H* using as columns the first two basis vectors, the eigenvalues of which constituted more than 95% of the total sum of the eigenvalues. Then the basis vectors were used to calculate principal components $\hat{\theta }$ for all subjects as in Equation (5).

(5) $$\hat{\theta }=ZH$$

Columns and rows of the matrix $\hat{\theta }$ correspond to principal components and subjects respectively. Thus, the two columns (principal components) of $\hat{\theta }$ consist of projections of the sEMG parameters on two directions of the greatest variance.

## 3. Results

sEMG parameters were statistically significantly different (*p* < 0.05) between the eight indivisible groups for nine combinations of loads and parameters. See Table 4 for the loads and parameters. The inter-group comparisons showed that sEMG parameters values for the P-AP/TA group were statistically significantly different from those for the P-AP/T, P-AP/A, and P-DN groups. The P-AP/T group had significantly lower values of DET, L, TT, and ENTR at 1 kg than the P-AP/TA group. The P-AP/A group had significantly lower values of L_max_ at 0 kg, DET, L, and ENTR at 1 kg and significantly higher values of DIV at 0 kg and SampEn at 1 kg than the P-AP/TA group. The P-DN group had significantly lower values of L and ENTR at 1 kg and significantly higher values of SampEn at 1 kg than the P-AP/TA group. See Table 5 for median values of the sEMG parameters. There were no statistically significant differences in any other inter-group comparisons of sEMG parameters. See Table 4 for the *p*-values from Mann–Whitney test of hypotheses H1–H10. Only hypotheses H4, H6, and H7 (comparisons with the P-AP/TA group) produced small *p*-values.

**Table 4:** ***p*****-values from Mann–Whitney test of hypotheses H1–H10**.

**Parameter**	**Load (kg)**	**H1**	**H2**	**H3**	**H4**	**H5**	**H6**	**H7**	**H8**	**H9**	**H10**
L_max_	0	0.767	0.605	0.65	1.04·10^−2^	0.849	2.46·10^−2^	4.68·10^−3^	0.833	0.267	0.7
DIV	0	0.767	0.605	0.65	1.04·10^−2^	0.849	2.46·10^−2^	4.68·10^−3^	0.833	0.267	0.7
SampEn	1	0.325	0.544	0.104	3.86·10^−3^	0.767	7.49·10^−3^	3.78·10^−3^	0.833	1	1
DET	1	0.793	0.669	0.451	8.62·10^−3^	0.375	2.41·10^−3^	9.91·10^−4^	1	0.833	1
L	1	0.555	0.77	0.19	4.75·10^−3^	0.291	1.79·10^−3^	1.97·10^−3^	0.667	0.667	1
TT	1	0.599	1	0.383	7.1·10^−3^	0.422	2.8·10^−3^	6.19·10^−3^	0.383	0.383	0.7
ENTR	1	0.646	0.911	0.302	4.75·10^−3^	0.278	1.53·10^−3^	2.57·10^−3^	0.667	1	1
L	2	0.218	0.981	0.882	1.24·10^−2^	0.944	2.06·10^−2^	6.98·10^−3^	0.667	0.833	1
V_max_	2	0.157	0.904	0.458	1.96·10^−2^	0.877	2.17·10^−2^	2.34·10^−2^	0.283	0.183	0.5

*Only sEMG parameters and loads combinations those were statistically significantly different in comparison of the eight indivisible patients groups using Kruskal–Wallis test (H0 in Table 3) are included*.

**Table 5:** **Comparisons of median values of sEMG parameters between P-AP/TA and other groups**.

**Parameter**	**Load (kg)**	**P-AP/TA**	**P-AP/A**	**P-AP/T**	**DN**	**Con**
L_max_ (ms)	0	25.5	10.8^*^	11	9.8	7.6
DIV (Hz)	0	39.2	92.4^*^	91.2	102.6	131.6
SampEn	1	0.818	0.869^*^	0.877	0.894^*^	0.877
DET	1	0.962	0.899^*^	0.849^*^	0.881	0.894
L (μs)	1	462	346^*^	309^*^	291^*^	325
TT (μs)	1	321	254	251^*^	252	253
ENTR (bit)	1	4.01	3.33^*^	3.12^*^	3.05^*^	3.30

* *p < 0.005 significant differences with the P-AP/TA group after Bonferroni correction. *p* < 0.05 for all comparisons with the P-AP/TA group shown in this table*.

The All-AP group had statistically significantly higher values of SAS and AIMS than the All-DN group. See Table 6 for the median values of SAS and AIMS. No correlations were detected between the sEMG parameters and the clinical scales in the All-All and All-AP groups.

**Table 6:** **Comparison of median values of SAS and AIMS**.

**Scale**	**All-DN**	**All-AP**
SAS	0	3^*^
AIMS	0	1^**^

* p = 9.05 · 10^−5^;

** *p = 0.0166 for the comparisons between the All-AP group and the All-DN group*.

**Figure 2:**
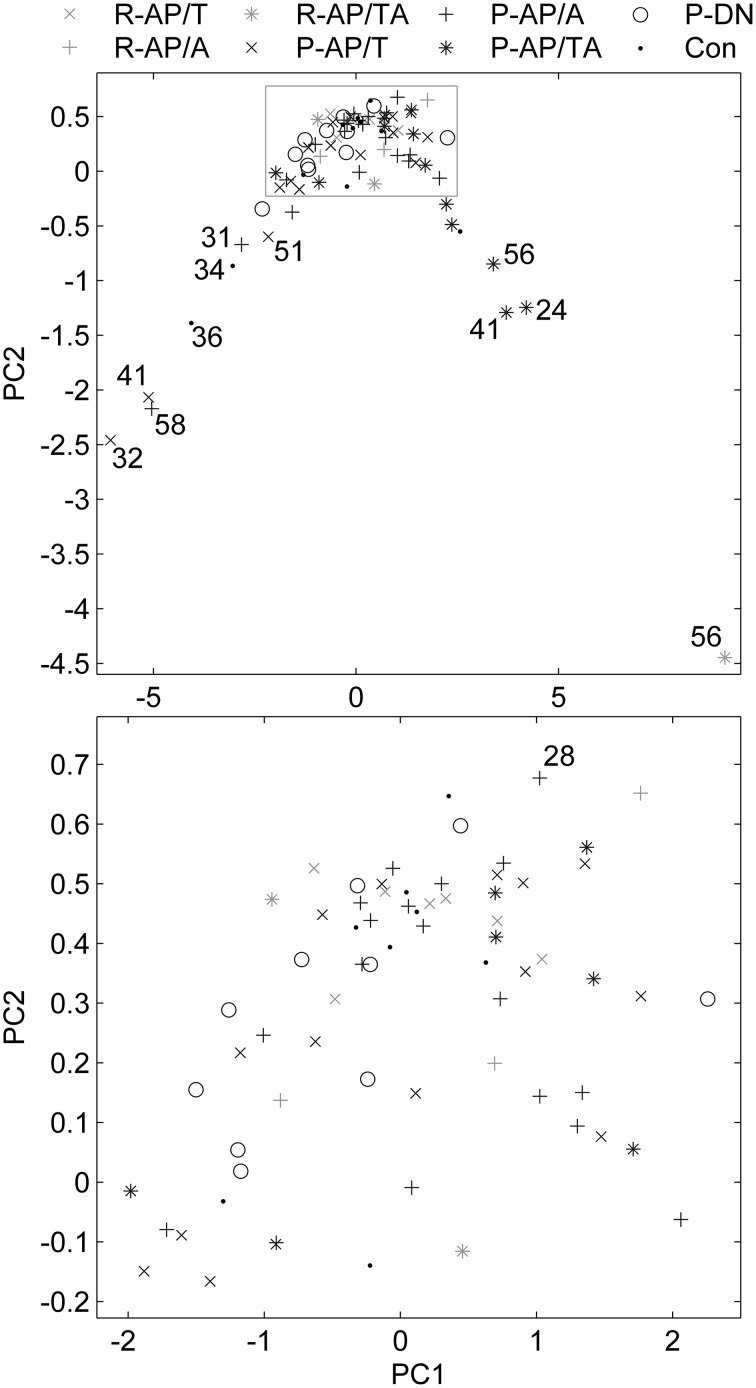
**Feature vectors of the subjects in a space of principal components**. Bottom plot is the gray rectangle on the upper plot. Numbers near the outliers are ages of the patients.

**Figure 3:**
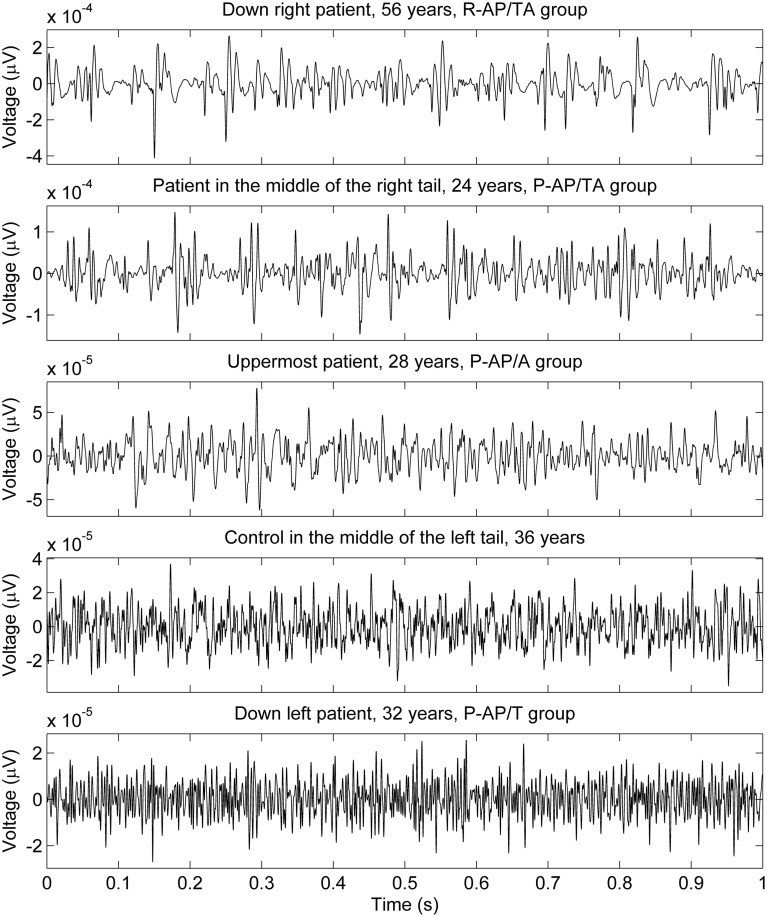
**sEMG of some subjects measured with 1 kg load on palm**. The locations of the subjects on the PC plot (upper part of Figure 2) are described in the graphs titles.

The data points composed a two-tailed curved distribution on the plot from PCA (Figure 2), which has first and second principal components as its *x*- and *y*-axes correspondingly. The All-AP/TA group tends to scatter around the right tail of the distribution. The P-DN group forms a tight cluster near the origin. Graphic changes of myoelectical signal are visible when moving from one tail of the distribution to another. See the graphs of sEMG of subjects located on various areas of the PC-plot on Figure 3.

## 4. Discussion

We hypothesized that sEMG parameters, especially the nonlinear ones, might correlate with the clinical symptoms and medication in SZ patients. We hoped to set a kind of “muscle channel” of information on SZ from the central nervous system via sEMG parameters. Earlier, we found that SZ patients in remission phase were undistinguishable from the control group by sEMG parameters, while those in psychosis phase had greater values of the nonlinear sEMG parameters, e.g., determinism (Miroshnichenko et al., 2014). Increased determinism of sEMG may evidence elevated degree of motor units activity synchronization (Fattorini et al., 2005). In turn, this may be indicative of the neuroleptic-induced parkinsonism (NIP), which is very common in SZ patients treated by antipsychotics (Friedman, 2014). Therefore, we expected presence of NIP in the All-AP group of patients.

In the present study, sEMG parameters in the controls were not different from those of the drug-naïve SZ patients who had never taken antipsychotics (P-DN, hypothesis H1). Thus, in contrary to this hypothesis (H1), “intact” SZ patients were electromyographically indistinguishable from the healthy controls. Thus, the SZ-related neurotransmitter disorders in the CNS do not affect the spinal cord. Absence of correlation between PANSS and sEMG parameters supports that statement.

However, we found that the SZ patients who were administered both typical and atypical antipsychotics (P-AP/TA) presented statistically significant difference of the sEMG parameters from the groups of drug-naïve patients and those receiving exclusively typical or atypical antipsychotics (hypotheses H4, H6, and H7 were rejected). In average, the nonlinear sEMG parameters were almost twice lower or higher for the P-AP/AT group of SZ patients in comparison to the other medication groups. It is known that atypical AP, unlike the typical ones, usually produce less NIP (Gurevich et al., 2012; Knol et al., 2012; Marras et al., 2012). We probably evidenced a kind of synergistic effect of these two classes of AP, i.e. our result may mean the greater blockade of the dopaminergic receptors in the CNS in case of combined typical/atypical AP therapy. It is likely that increased synchronization of MU activity may account for elevated regularity of sEMG in the P-AP/TA patients (Fattorini et al., 2005). However, an alternative explanation may be figured out. It is known that in SZ patients the denervation/reinnervation process takes place (Crayton et al., 1977). Thus, a large (giant) motor unit might have been firing constantly in the proximity of the recording electrode due to sprouting effect (Seburn et al., 1996).

In a whole, the sEMG data proved to be indicative of the elevated regularity of the myoelectrical signal in the group of SZ patients who were administered both typical and atypical AP. This would allow discriminating groups of SZ patients those differ by their anti-SZ medication using sEMG parameters. An example of sEMG with appreciably elevated regularity can be seen on the topmost graph of Figure 3. The difference of SAS and AIMS between the group of patients who were taking any antipsychotics at the moment of study and the group of drug-naïve patients (hypotheses H11 and H12 were rejected) supports the hypothesis about presence of NIP in the All-AP group.

It can be seen on the PCA plot that most of outliers in the right tail of the data distribution are aged SZ patients. This prompts that the age, in addition to the disease *per se*, may contribute to the sEMG characteristics in SZ, even though the number of the points is not enough for any reliable conclusions. It is known that the motor system undergoes substantial age-related remodeling (Jang and van Remmen, 2011). sEMG parameters are also modified throughout the life (Boccia et al., 2014). This suggests further investigation of sEMG in aged SZ patients.

In the present study, the best discrimination power between groups was the characteristic of smaller loading of arms (no loading and 1 kg). That might be attributable to a known fact that, for example, in the Parkinson's disease patients sEMG data becomes more “normal” under bigger loads. This probably evidenced emergency of a “normal” physiological muscle tone evoked by an external load (Meigal et al., 2009). Thus, smaller loads seem to be more informative in sEMG-based diagnostics.

In conclusion, the nonlinear sEMG parameters might be helpful in early detection of neuroleptic-induced parkinsonism during treatment with typical and atypical antipsychotics. Further studies are needed to elucidate the possible role of the disease duration, age and sex in forming sEMG characteristics in SZ.

## Author contributions

AM has contributed by the research conception and design of the work, data acquisition and interpretation, and by writing of the manuscript. GM has contributed by data analysis and interpretation, and by writing of the manuscript. AK has contributed by design of the work and data acquisition and interpretation. SD, SR, and PK have contributed by data analysis and critical revision of the manuscript. All the authors have read and approved the manuscript before the publication and agree to be accountable for all aspects of the research.

### Conflict of interest statement

The authors declare that the research was conducted in the absence of any commercial or financial relationships that could be construed as a potential conflict of interest.
